# State of the Art: Biomaterials and Oral Implantology—Editorial

**DOI:** 10.3390/jfb17100518

**Published:** 2026-10-15

**Authors:** Markel Diéguez-Pereira, Aritza Brizuela-Velasco

**Affiliations:** 1 Faculty of Health Sciences, Miguel de Cervantes European University, 47012 Valladolid, Spain; 2 DENS-ia Research Group, Faculty of Health Sciences, Miguel de Cervantes European University, 47012 Valladolid, Spain

## 1. Introduction

This Special Issue of the *Journal of Functional Biomaterials* brings together five original articles on the materials, designs and diagnostic tools that support the repair and replacement of oral tissues. Dental implants and removable prostheses are now routine treatments, yet their long-term success still depends on how biomaterials interact with bone and soft tissue, how they behave under masticatory load, and how clinicians detect early failure.

The contributions span the full translational pathway. They include an in vitro biological study of a new implant coating, an in vitro wear study of artificial denture teeth, a finite element analysis of peri-implant bone, an in vitro diagnostic study of resonance frequency analysis, and a retrospective clinical cohort with a follow-up of at least five years. Together, they show how laboratory, computational and clinical evidence can inform one another in dental biomaterial research.

## 2. Overview of the Published Articles

**Implant surface biology.** Alex et al. [1] evaluated a coating of zirconia doped with 5% calcium (Ca-SZ), deposited by physical vapour deposition on TA6V titanium alloy. Compared with uncoated TA6V and zirconia-based reference materials, Ca-SZ improved the viability and proliferation of Saos-2 osteoblast-like cells and HaCaT keratinocytes over 96 h. It also lowered TNF-α and IL-6 release. After 21 days in osteogenic conditions, Saos-2 cells on Ca-SZ produced more soluble collagen, alkaline phosphatase, osteopontin and osteocalcin, and formed hydroxyapatite crystals confirmed by SEM and EDS. The authors propose Ca-SZ as a candidate surface for hybrid implants, although these findings, based on three biological replicates, now need confirmation in vivo.

**Prosthetic materials and wear.** Yelekçi et al. [2] measured the wear of isosit and nanohybrid composite artificial teeth against three antagonists, using a chewing simulator (600,000 cycles) and 3D volumetric scanning. Porcelain antagonists produced roughly twice the wear of isosit or nanohybrid antagonists (*p* < 0.05), whereas the two artificial tooth materials performed similarly. Identical pairings across the arches showed a non-significant trend towards less wear. The study supports avoiding porcelain against resin-based denture teeth in removable prostheses.

**Biomechanics of implant placement.** Ceddia et al. [3] used finite element analysis to study how inter-implant distance (3, 1.5 and 1 mm) affects stress in D3 and D4 bone around conometric implants placed at crestal or subcrestal level. Cortical stress rose as the distance decreased, most markedly with crestal placement: in D3 bone, from 7.2 MPa at 3 mm to 16.5 MPa at 1 mm, and close to the 20 MPa remodelling threshold in D4 bone. Subcrestal placement lowered cortical stress in both bone qualities. The authors rightly stress that these are comparative computational results and do not establish the clinical safety of a 1 mm inter-implant distance.

**Diagnosis of marginal bone loss.** Pérez-Pevida et al. [4] tested whether resonance frequency analysis (RFA) can detect peri-implant bone loss. Forty bone-level implants were placed in polyurethane blocks, and a circumferential defect was then created by removing the cortical portion around each implant. The mean implant stability fell from 72.2 to 63.3 ISQ, a statistically significant decrease. RFA may therefore help monitor marginal bone, but in vivo and longitudinal studies are needed to define thresholds for early detection.

**Clinical outcomes of prosthetic retention.** Cumpătă et al. [5] analysed 528 bone-level implants placed with maxillary sinus floor elevation in 248 patients, with CBCT follow-up of at least 60 months. The mean marginal bone level at the final follow-up was 0.133 mm for cement-retained and 0.105 mm for screw-retained restorations. In the primary analysis, which used generalised estimating equations to account for clustering within patients, definitive retention mode was not associated with marginal bone level (B = −0.0028 mm; *p* = 0.684), nor with its change over time. The authors carefully note that these observational data show neither superiority nor equivalence, and that retention should be chosen according to the wider prosthetic context.

## 3. Common Themes and Future Directions

Three themes run through the collection. First, preserving marginal bone is a shared endpoint: it is addressed biologically by a coating that supports osteoblast differentiation, mechanically by placement depth and inter-implant distance, diagnostically by RFA, and clinically by prosthetic retention mode. Second, subcrestal placement emerges as a recurring question, with computational data suggesting lower cortical stress that still awaits clinical confirmation. Third, several contributions show welcome methodological caution, distinguishing statistical from clinical relevance and avoiding claims of equivalence from non-significant results.

The main limitation of the collection is that four of the five studies are preclinical. In vitro, polyurethane and finite element models allow controlled comparisons, but they cannot reproduce the biological variability, loading patterns and microbiota of the oral environment. Future work should test promising materials such as calcium-doped zirconia in animal and clinical studies. It should also validate diagnostic thresholds for RFA against radiographic bone loss in patients, and use prospective randomised designs that account for the clustering of implants within patients.

## 4. Concluding Remarks

The five articles in this Special Issue illustrate how advances in dental biomaterials depend on combining materials science, biomechanics, diagnostics and clinical follow-up. We thank all the authors for their contributions, the reviewers for their careful and constructive work, and the editorial staff of the *Journal of Functional Biomaterials* for their support. Contribution 4 was co-authored by the Guest Editors and was handled independently by another Academic Editor of the journal.

## Data Availability

No new data were created or analysed in this study. Data sharing is not applicable to this article.
